# Ten-year risk of fatal cardiovascular disease and its association with metabolic risk factors among waste pickers in South Africa

**DOI:** 10.1186/s12872-021-02150-y

**Published:** 2021-07-10

**Authors:** Felix Made, Engelbert A. Nonterah, Nonhlanhla Tlotleng, Vusi Ntlebi, Nisha Naicker

**Affiliations:** 1 Epidemiology and Surveillance Section, National Institute for Occupational Health, National Health Laboratory Service, Johannesburg, 2000 South Africa; 2 Faculty of Health Sciences, School of Public Health, University of Witwatersrand, Johannesburg, 2000 South Africa; 3 Navrongo Health Research Centre, Ghana Health Service, Navrongo, Ghana; 4 Julius Global Health, Julius Centre for Health Sciences and Primary Care, University Medical Centre, Utrecht University, Utrecht, The Netherlands; 5 Department of Environmental Health, Faculty of Health Sciences, University of Johannesburg, Johannesburg, 2000 South Africa

**Keywords:** Obesity, Hypertension, Diabetes, Cholesterol, Smoking, Age, Understudied populations

## Abstract

**Background:**

Cardiovascular disease (CVD) is the leading cause of death among non-communicable diseases in South Africa. Several metabolic risk factors contribute to the development of CVD. Informal workers such as waste pickers could be unhealthy lifestyle naive, and most public health research on CVD does not include this understudied population. This study estimated the 10-year risk of fatal CVD and its association with metabolic risk factors in an understudied study population of waste pickers in Johannesburg, South Africa.

**Methods:**

A cross-sectional survey was conducted among waste pickers in two landfill sites in Johannesburg. We used the Systematic Coronary Risk Evaluation (SCORE) risk charts to estimate the 10-year risk of fatal CVD. We then employed ordinary least squares regression to assess the association between the 10-year risk of fatal CVD with metabolic risk factors. Other variables adjusted in the regression model were HIV status, education, income, injuries from work, clinic visits in the previous 12 months, and alcohol consumption.

**Results:**

A total of 370 waste pickers were included in this analysis, 265 (73.41%) were males. The mean age of the participants was 34 years. The majority were between the age of 20 and 39 years. More than 55% of the waste pickers did not visit a clinic in the previous 12 months, and 68.57% were smoking. The 10-year survival probability from CVD was more than 99% for both males and females. In the multivariable regression model, elevated blood glucose showed a non-significant increase in the mean percentage of 10-year risk of fatal CVD. Waste pickers who were overweight/obese, and hypertensive had high statistically significant mean percentages of the 10-year risk of fatal CVD compared to those who did not have the metabolic risk factors.

**Conclusions:**

Prevention of 10-year risk of fatal CVD in this understudied population of waste pickers should target the control of obesity, hypertension, and diabetes. Health awareness and education for waste pickers will be an important step in reducing the burden of these metabolic risk factors. We further recommend that health systems should recognize waste pickers as a high-risk group and consider extensive CVDs surveillance.

**Supplementary Information:**

The online version contains supplementary material available at 10.1186/s12872-021-02150-y.

## Background

The mortality rates of non-communicable diseases (NCDs) are higher than that of HIV/AIDS, of which cardiovascular disease (CVD) is the leading cause in South Africa [1, 2]. CVDs are a group of disorders of the heart and the blood vessels, which include coronary heart disease (CHD), stroke, and other vascular diseases. According to the World Health Organization (WHO), more people die annually from CVD than any other disease [3]. The WHO further reported that in the year 2016, about 17.9 million people died from CVD, which accounted for 31% of all global deaths. Over 80% of CVD deaths take place in low- and middle-income countries.

The World Heart Federation (WHF) initiated a CVD prevention and control program called 25 by 25 Global Target. This initiative aimed to reduce the premature mortality rate from CVD by 25% in 2025 [4]. In South Africa, various efforts including a plan to reduce NCDs by 25% by 2020 was set alongside increased taxation of sugar and tight tobacco legislation [1, 5]. Other efforts have been placed on improved treatment of cardiovascular diseases in South Africa and identification of risk factors [6].

A rapid rise in development and urbanization has led to many people practising unhealthy lifestyles, which increases the prevalence of metabolic risk factors. These risk factors include high body mass index (BMI), raised blood pressure, blood glucose, and elevated cholesterol level [3, 7]. The metabolic risk factors are the major determinants of CVD according to the WHO [3]. Underlying modifiable risk factors such as tobacco use, alcohol abuse, physical inactivity, and a poorly balanced diet could increase the risk of developing CVD among waste pickers, where unhealthy lifestyle practices are probably high. A high unemployment rate (26.7%) and increased urbanization, are associated with a rise in the informal economy, such as waste recycling in South Africa (SA) [8]. Waste pickers contribute to the economy and waste recycling efforts. An estimated 1.5 million tonnes of wastes are dumped in landfill sites in Johannesburg [9].

Work environments such as landfill sites have several health hazards such as noise, work stress, chemicals and pollutants, and increased workload. These health hazards were associated with the development of CVD in previous literature [10–12]. Despite the efforts put in place to reduce the prevalence and incidence of CVD, waste pickers are most likely marginalized within the healthcare system, yet their CVD risk could be very high. The medical recommendation for the prevention of CVD needs to be based on an assessment of the “individual’s total risk burden” not on a single risk factor [13, 14]. There is evidence that risk factors tend to occur in clusters [15], and the majority of the individuals have more than one risk factor, which when combined leads to increased fatal CVD risk [16]. As a result, the use of the CVD risk score as the first line of prevention among people who do not clinically indicate CVD symptoms is highly recommended [17]. This study aimed to estimate the 10-year risk of fatal CVD and assessed its association with metabolic risk factors among an understudied population of waste pickers in Johannesburg, South Africa.

## Methods

### Study design and setting

This study was a cross-sectional survey conducted in two of the largest landfill sites in Johannesburg. The landfill sites were closer to densely populated areas, with the largest number of waste pickers in Johannesburg. One of the study sites had approximately 600 waste pickers and the other had approximately 3000 waste pickers.

### Study population and sample

The study population included male and female waste pickers aged 18 years or older. A convenience sampling frame was employed to include waste pickers in the study. All waste pickers who were available on the day of data collection were approached. The study aim was explained to each one of them in a language they understand. Those who agreed to a written consent were included in the study.

### Data collection tools and methods

A structured questionnaire was translated into languages spoken by individual waste pickers. Data were imputed directly into the RedCap database using the electronic version of the structured questionnaire (see Additional file 1). The research was conducted according to the ethical principles in the declaration of Helsinki (1964). Detailed ethics statement is in the declarations section of the manuscript.

The 10-year risk of fatal CVD was calculated using a comparable method from the European based Systematic Coronary Risk Evaluation (SCORE) project [18]. We opted to use the SCORE methods because the Framingham data, an analyses strategy for 10-year risk of fatal CVD tended to overestimate absolute risk in populations with lower coronary heart disease rates [19, 20]. In this study, we assumed that the waste pickers have low-risk CVD which is comparable to the low-risk countries reported in the SCORE risk function. This is because they constantly do physical activities, a major factor for the development of CVD. The physical activities may include strenuous work in pulling, pushing, bending, and walking during the collection of waste materials. Also, the SCORE risk function allows for variation in the risk of CVD [18].

The risk factors used in the SCORE risk function are widely regarded as the classical CVD risk because of their strong association with CVD. Therefore, we used the SCORE low CVD risk estimates based on sex, age, systolic blood pressure, total cholesterol, and current smoking status. We took anthropometric measurements for weight and height to generate BMI (weight in kilograms per height in meters squared) category: underweight was defined as less than 18.5), the normal weight started from 18.5 to < 25), overweight (25 to < 30), and obesity from 30 and above [21]. Systolic and diastolic blood pressure was measured three times, and the average of the three measurements was taken to generate the final variables. Hypertension was defined as systolic blood pressure > = 140 mmHg and/or diastolic blood pressure > = 90 mmHg [21]. Point of care testing was done for random blood glucose. Random blood glucose was used as a proxy to indicate possible diabetes without the diagnosis. The random blood glucose was categorized into three groups with 4.5–7.8 mmol/L as normal and 7.8–11.1 mmol/L,  > 11.1 mmol/L as moderate and high, respectively [22]. Also, total cholesterol in mmol/L was measured. Other self-reported data collected were for education, injuries, clinic visits, alcohol, average monthly income, and HIV.

### Statistical analysis

Statistical analysis was undertaken using STATA SE version 15.1 (4905 Lakeway Drive, College Station, TX, USA). Descriptive statistics for continuous variables were summarized as means and standard deviations, factor variables were presented as frequencies and percentages. We used the SCORE method to estimate the 10-year risk of fatal CVD [18]. The cardiovascular risk functions were calculated using a Weibull proportional hazards model for baseline survival function only. The hazard function for men and women were calculated separately, and the risk factor estimate was done for combined sexes. The hazard function calculation was based on the participant's age, to produce an estimate that depends on the observed age rather than using the length of the study period as in the traditional survival function. The 10-year risk of fatal CVD was based on the conditional probability of death in the next 10 years given a participant’s current age. The risk of death was estimated by combining two separate models, one for coronary heart disease (CHD) and another for non-coronary heart disease (non-CHD).

To calculate the 10-year risk for fatal cardiovascular disease, we first estimated the underlying risk for CHD and non-CHD. This was done separately for participant's current age and their age in 10 years.

The survival probability is:

1 $$\begin{aligned} {\text{S}}_{0} \left( {{\text{age}}} \right) & = {\text{exp}}\left\{ { - \left( {{\text{exp}}\left( \alpha \right)} \right) \, \left( {{\text{age}} - 20} \right)^{p} } \right\} \\ {\text{S}}_{0} \left( {{\text{age}} + {1}0} \right) & = {\text{exp}}\left\{ { - \left( {{\text{exp}}\left( \alpha \right)} \right)\left( {{\text{age}} - 10} \right)^{p} } \right\}* \\ \end{aligned}$$

where α and *p* are low-risk constant coefficients available in the SCORE report. * Weibull model is expressed as λ = exp(α).

We then calculated the weighted sum, *w*, of risk factors including cholesterol, smoking, and systolic blood pressure, this was also done separately for CHD and *non-CHD.*

2 $$w = \, \beta_{chol} \left( {{\text{Cholesterol}} - 6} \right) + \beta_{SBP} \left( {{\text{SBP}} - 120} \right) + \beta_{smoker} \left( {{\text{current}}} \right).$$

where β is a constant coefficient available in the SCORE report [18].

To get the probability of survival at each age for each cause, we combined the underlying risk for CHD and Non-CHD disease at current participants' age and age in 10 years in Eq. 1, with the weighted sum of participant’s risk factors in Eq. 2.

$$\begin{aligned} {\text{S}}\left( {{\text{age}}} \right) & = [\left\{ {{\text{S}}_{0} \left( {{\text{age}}} \right)} \right\}^{{{\text{exp}}(w)}} \\ {\text{S}}\left( {{\text{age}} + {1}0} \right) & = \left\{ {{\text{S}}_{0} \left( {{\text{age}} + {1}0} \right)} \right\}^{{{\text{exp}}(w)}} \\ \end{aligned}$$

The 10-year survival probability based on a participant's current age and age in 10 years is:

$${\text{S}}_{{{1}0}} \left( {{\text{age}}} \right) = {\text{S}}\left( {{\text{age}} + {1}0} \right)/{\text{S}}\left( {{\text{age}}} \right)$$

The 10-year risk for each point is given by:

$${\text{Risk}}_{10} = 1 - {\text{S}}_{10} \left( {{\text{age}}} \right)$$

Finally, we combined the risk for CHD and non-CHD to produce the 10-year risk of fatal CVD.

$${\text{CVDRisk}}_{10} \left( {{\text{age}}} \right) = \left[ {{\text{CHDRisk}}\left( {{\text{age}}} \right)} \right] + \left[ {{\text{Non-CHDRisk}}\left( {{\text{age}}} \right)} \right]$$

We then used ordinary least squares regression to assess the association between the 10-year risk of fatal CVD and known metabolic risk factors. To avoid, any spurious findings, we adjusted our model with full variables inclusion and systematic reduction. Separate models were fitted for continuous variables that have been transformed to factor variables, with all other covariates adjusted. We opted to use the bootstrapping technique with maximization of variance–covariance estimation on the log-transformed 10-year risk of the fatal CVD response variable. The bootstrapping model produced bias-corrected 95% confidence intervals from the underlying unobserved clustering from the data.

## Results

A total of 370 waste pickers were included in this study, of which 96 (26.59%) were females (Table 1). The mean age of the participants was 34 years. On average the waste pickers worked for 6.7 years at the landfill sites. The participants on average earned more than 117.24 United States Dollars monthly. The majority of the participants were in the age group 20 to 39 years old. More than 79% had reached at least a secondary education level. There were 146 (41.24%) waste pickers who at least visited a clinic in the previous year. The number of those currently smoking was 240 (68.57%). A proportion of 41.73% of the waste pickers was currently consuming alcohol consumption. At least 80% of the waste pickers had some sort of injury from sharp objects or any form during their work at the landfill sites. The majority (88.41%) of them were HIV negative. Most of the waste pickers were of normal weight 202 (54.89%), non-hypertensive 288 (77.84%), and had normal random blood glucose level 362 (97.84%).

**Table 1:** Description of demographic, lifestyle, and metabolic risk factors

*Continuous variables*	Mean (standard deviation)
Age (years)	34.00 (10.00)
Years of work	6.70 (5.50)
Income (United States Dollars)	123.58 (136.05)
BMI (kg/m^2^)	22.71 (6.59)
Blood glucose (mmol/L)	5.10 (2.43)
Total cholesterol (mmol/L)	3.23 (1.33)
Systolic blood pressure (mmHg)	120.30 (21.31)
Diastolic blood pressure (mmHg)	76.46 (17.29)
*Factor variables*	n (%)
Sex	
Male	265 (73.41)
Female	96 (26.59)
Age group	
Less than 20	6 (1.62)
20–29	139 (37.57)
30–39	136 (36.76)
40–49	38 (10.27)
50+	51 (13.78)
Education	
None	15 (4.17)
Primary	59 (16.39)
Secondary	286 (79.44)
Clinic visits	
No	208 (58.76)
Yes	146 (41.24)
Smoking status	
No smoking	110 (31.43)
Currently smoking	240 (68.57)
Alcohol	
No	162 (58.27)
Yes	116 (41.73)
Injuries	
No	62 (17.27)
Yes	297 (82.73)
HIV	
No	305 (88.41)
Yes	40 (11.59)
BMI category	
Underweight	83 (22.55)
Normal weight	202(54.89)
Overweight	40 (10.87)
Obese	43 (11.68)
Hypertension	
Non-hypertensive	288 (77.84)
Hypertensive	82 (22.16)
Blood glucose category	
Normal	362 (97.84)
Moderate	4 (1.08)
High	4 (1.08)

Table 2 presents the distribution of participants, the 10-year risk of fatal CVD, and the 10-year survival probability from CVD by sex. The 10-year risk of fatal CVDs increased with increasing age in both sexes. Waste pickers at the age of 50 or more had a high 10-year risk of fatal CVDs, which had a slightly higher average in males (1.62) than females (1.22). The majority of the participants had normal weight in both males and females. Waste pickers that had a normal weight revealed a high 10-year risk of fatal CVD followed by overweight participants, especially in females. Females that indicated a high glucose level or were hypertensive, and males that were hypertensive recorded a high 10-year fatal CVD risk. A male participant who was categorized with high blood glucose concentration had a lower 10-year risk of fatal CVD than those with normal or moderate blood glucose levels. The 10-year survival probability of CHD and non-CHD were similar at more than 99% probability (the chance of not dying from CVD) of survival from any fatal CVD events.

**Table 2:** The distribution of age group, the 10-year risk of fatal CVD, and 10-year survival probability by sex

	Male		Female	
	n (%)	10-year CVD risk Mean (SD)	n (%)	10-year CVD risk Mean (SD)
Variables		0.130 (0.584)		0.340 (2.209)
Age group (years)				
Less than 20	5 (1.89)	–	1 (1.04)	–
20–29	123 (46.42)	0.004 (0.004)	16 (16.67)	1.7 × 10^−5^ (1.5 × 10^−5^)
30–39	100 (37.74)	0.038 (0.036)	36 (37.60)	0.007 (0.012)
40–49	21 (7.92)	0.301 (0.328)	17 (17.71)	0.062(0.048)
50+	16 (6.02)	1.621 (1.811)	26 (27.08)	1.220 (4.216)
BMI category				
Underweight	70 (26.62)	0.139 (0.727)	13 (13.54)	0.035 (0.118)
Normal weight	147 (55.87)	0.155 (0.599)	50 (52.08)	0.559 (3.119)
Over weight	24 (9.13)	0.102 (0.174)	14 (14.58)	0.209 (0.272)
Obese	22 (8.37)	0.069 (0.159)	19. (19.79)	0.115 (0.204)
Blood glucose category				
Normal	263 (99.27)	0.141 (0.587)	91 (94.79)	0.340 (2.270)
Moderate	1 (0.38)	0.002 (–)	2 (2.08)	0.311 (0.419)
High	1 (0.38)	0.038 (–)	3 (3.13)	0.368 (0.449)
Hypertension				
Non-hypertensive	216 (81.51)	0.077(0.425)	65 (67.71)	0.074 (0.157)
Hypertensive	49 (18.49)	0.451(1.019)	31 (32.29)	0.872(3.806)
		10-year survival probability of CVD		
CHD	–	99.82% (0.10%)	–	99.80% (0.10%)
Non-CHD	–	99.90% (0.04%)	–	99.70% (1.71%)

*SD* standard deviation

Table 3 presents the association between the 10-year risk of fatal CVD and metabolic risk factors. BMI and hypertension were independently associated with a 10-year risk of fatal CVD after adjusting for HIV, income, educational status, number of years of work, clinic visits in the past months, injuries at work, and alcohol consumption. For a unit increase in BMI, there was a 2.53% average increase in 10-year risk fatal CVD events. Overweight participants had a statistically significant increase in the 10-year risk of fatal CVD (mean: 133.96%; 95% CI: 1.10%; 493.57%). Waste pickers who were obese also had a 20.92% increase in the 10-year risk of fatal CVD events but the difference was not significant compared to those who were underweight. There was a 469.73% mean increase in the 10-year risk of fatal CVD with a statistically significant difference compared to the normal or non-hypertensive individuals. Blood glucose was not significantly associated with a 10-year risk of fatal CVD in the study population.

**Table 3:** Association between the 10-year risk of fatal CVD and metabolic risk factors

Factors	Mean (%)	LCL( %)	UCL (%)
BMI	2.53	− 0.69	5.44
BMI category			
Underweight	0		
Normal	19.72	− 32.63	107.71
Overweight	133.96	1.10	493.57
Obese	20.92	− 45.61	192.99
Blood glucose	4.08	− 8.24	9.85
Blood glucose category			
Normal	0		
Moderate	− 2.95	− 83.56	586.20
High	343.70	− 22.66	1064.64
Hypertension status			
Non-hypertensive	0		
Hypertensive	469.73	174.01	1049.60

Confidence intervals that do not include zero is statistically significant

Adjusted for HIV, income, education status, number of years of work, clinic visits in the past 12 months, injuries at work, and alcohol consumption

*LCL* lower confidence limit, *UCL* upper confidence limit

## Discussion

The prevention of CVD including heart attacks and stroke should be based on a cardiovascular risk assessment which is better than treatment based on a single risk factor [23]. This is largely because CVD risk factors tend to occur in clusters and have pronounced fatal effects than single risk factors [24, 25]. The 10-year risk of fatal CVD and associated diseases of metabolic risk factors that arise from lifestyle choices among the waste pickers have not been studied to our knowledge. Our study aimed to estimate the 10-year risk of fatal CVD and its association with metabolic risk factors in this largely unhealthy lifestyle naive and vulnerable population.

The average 10-year risk of fatal CVD among female waste pickers was higher than that of males. This is inconsistent with previous literature that showed CVD is more common overall in men than women, due to men's risky lifestyle choices [26, 27]. Our finding suggests differences in age, women waste pickers were generally older than men. In addition, the CVD risk could have been increased by lifestyle diseases including high blood glucose, hypertension, and obesity which tend to be more prevalent in women in this study. Our study indicated that increasing age increases the 10-year risk of fatal CVD in both men and women, this is in line with a previous report where CHD, a major CVD, increased with increasing age in women and men, and even more common in individuals older than 50 years [24]. In another study, men had almost doubled the 10-year risk for CVD compared to women at both baseline and 1-year follow-up [28].

This study found that the mean of 10-year CVD risk was higher among normal weight than underweight, overweight and obese participants. There could be a metabolically obese normal weight population that is categorised as normal weight in this study, because of increased distribution of visceral fat and other metabolic conditions such as dyslipidaemia [29]. Although our study did not investigate, this finding is consistent with a report from a previous study which found that normal weight with central obesity was a risk factor for CVD [30]. Therefore, some of the normal-weight participants might have had central obesity.

We also observed that male participants with a high blood glucose level had a lower 10-year risk of fatal CVD than those who had normal or moderate blood glucose levels. This might be attributed to the knowledge of the disease which comes with healthy lifestyle choices in certain individuals. Hence other CVD-related risk factors are adequately managed. Furthermore, glucose levels could be generally low in this understudied population as has been reported in other African populations [31, 32]. The effect on CVD risk may result from genetic and gene-environment interactions. This observation in our study may also be due to unmeasured factors such as physical activity and diet that might have mediated or confounded the association between glucose and CVD risk.

The contradictory findings of the low and high CVD risk among participants with high blood glucose and normal weight, respectively, can be attributed to the sample size. The high glucose was found in just four waste pickers who probably did not have other risk factors for CVD which included smoking, advanced age, diabetes and high cholesterol level. The normal weight population were the highest in this study. This high population could also mean the majority were in advanced age and had other CVD risk factors including hypertension, diabetes, and dyslipidaemia [3, 33].

A high mean percentage increase in 10-year risk of fatal CVD events was associated with waste pickers who were overweight or obese, high blood glucose, and hypertensive after adjusting for other variables. According to the WHO, hypertension, increased blood glucose (diabetes) and obesity are the major metabolic risk factors for the development of CVD. Studies conducted on Brazilian waste pickers found that hypertension, diabetes, overweight, and obesity were very prevalent [34, 35]. This may imply that these metabolic risk factors are common in waste pickers even if they are of different geographic locations and share different cultural practices. Social, economic, and cultural change and poverty including stress were reported to be the drivers of these lifestyles diseases [3]. The waste pickers could be affected the worst given their level of poverty and stress, as they strive on daily basis to earn a living. Also, waste pickers might have no or rare access to healthcare, as reported previously [36]. Furthermore, another study reported that 37% of the waste pickers did not consult a doctor in more than a year and 8% never measured their blood pressure including other health measurements [34].

One of the limitations of this study was the small sample size which could have inflated the findings from this study and thus hide the potentially significant effect. The SCORE risk function was not validated in the black population, thus it may under or over-estimate 10-year CVD risk. There is a lack of data on exposures including noise, chemicals, pollutants and diet which could be risk factors for CVD, therefore, these findings should be interpreted with caution. The inclusion of waste pickers from 18 years old unlike in previous studies where only individuals from 40 years and older, could contribute to the low risk of fatal CVD [18, 37, 38]. Despite these limitations, our study does offer some insights on the 10-year risk of fatal CVD in a largely understudied population. It will serve as the basis to establish a large scale cohort of waste pickers for further research.

## Conclusions

Prevention of 10-year risk of fatal CVD in this understudied population of waste pickers should target the control of obesity, hypertension, and diabetes. Therefore, health awareness and education for waste pickers will be an important step in reducing these metabolic risks. We further recommend that health systems should recognize waste pickers as a high-risk group and consider extensive CVDs in near future.

## Supplementary Information

**Additional file 1.** Study Questionnaire for CVD in Waste Pickers.

## Data Availability

The datasets used and/or analysed during the current study are available from the corresponding author on reasonable request.

## Abbreviations

NCD: Non-communicable diseases
CVD: Cardiovascular diseases
SCORE: Systematic Coronary Risk Evaluation
BMI: Body mass index
