# Supplementary material for: Ten-year risk of fatal cardiovascular disease and its association with metabolic risk factors among waste pickers in South Africa
Source: BMC Cardiovasc Disord. 2021 Jul 10;21:336. doi: 10.1186/s12872-021-02150-y (PMC8272349; doi:10.1186/s12872-021-02150-y)
Supplement: Supplementary file 1 — Additional file 1. Study Questionnaire for CVD in Waste Pickers. [file 12872_2021_2150_MOESM1_ESM.pdf]

Waste pickers

Please complete the survey below.

Thank you!

Study ID	______________________
Consent obtained	◯ Yes<br>◯ No
Reason consent not obtained	◯ Refused<br>◯ Not enough time<br>◯ Language barrier
Date of interview	______________________
Name of interviewer	______________________
Name of landfill site	◯ Goudkoppies<br>◯ Marie Louise
Respondents' suburb	______________________

Demographic and Household Info.

Age	______________________
Gender	◯ Male<br>◯ Female
Main language spoken	◯ English<br>◯ Afrikaans<br>◯ Zulu<br>◯ Xhosa<br>◯ Ndebele<br>◯ Sepedi<br>◯ Sesotho<br>◯ Setswana<br>◯ Tsonga<br>◯ Swati<br>◯ Venda<br>◯ Other
Specify Other language	______________________

What is the level of education obtained	◯ No schooling<br>◯ Primary school<br>◯ Secondary school<br>◯ Tertiary level
Country of birth	◯ South African<br>◯ Other
If other, please name country	__________________________
If other, years lived in S.A	__________________________
Province of birth	◯ Gauteng<br>◯ Free State<br>◯ North West<br>◯ Eastern Cape<br>◯ Northern Cape<br>◯ Western Cape<br>◯ Mpumalanga<br>◯ Limpopo<br>◯ KwaZulu-Natal
How long have you lived in JHB?	__________________________
Where did you live before moving to JHB	__________________________
Why did you move to JHB?	________________________________
Do you live on or adjacent to a landfill?	◯ Yes<br>◯ No
Do you live in a:	◯ Formal house<br>◯ Informal dwelling<br>◯ Back yard dwelling/room - formal<br>◯ Back yard dwelling/room - informal<br>◯ Other (e.g. veld, bushes, street)
What do you mainly use for cooking at home?	◯ Electricity<br>◯ Paraffin<br>◯ Gas<br>◯ Wood<br>◯ Coal<br>◯ Other
What do you mainly use for heating at home?	◯ Electricity<br>◯ Paraffin<br>◯ Gas<br>◯ Wood<br>◯ Coal<br>◯ Other

Where do you mainly get your water from at home?	◯ River/stream<br>◯ Household tap<br>◯ Communal tap<br>◯ Borehole<br>◯ Other
Do you have access to toilet facilities where you live?	◯ Yes<br>◯ No
If yes, what type of toilet?	◯ Private flush toilet<br>◯ Communal flush toilet<br>◯ Pit latrine<br>◯ Chemical toilet<br>◯ Other
Do you have access to a place to wash yourself/hands at home?	◯ Yes<br>◯ No
How many people are there in your household?	__________________________________
How many adults (15 yrs and older)	__________________________________
how many children (14 yrs and younger)	__________________________________
Are you the sole bread winner for your household?	◯ Yes<br>◯ No
How many people do you support financially?	__________________________________
What is your average monthly income?	__________________________________
Do you receive any grants?	◯ Yes<br>◯ No
If yes, please indicate	☐ Pension<br>☐ Disability<br>☐ Child support
How much do you spend on Food every month?	__________________________________
How much do you spend on Transport (include taxi fare, petrol, car instalment) monthly	__________________________________
How much do you spend on Transport of waste where you sell it monthly?	__________________________________
How much do you spend on housing (rent, bond, etc) monthly	__________________________________

How much do you spend on Water monthly	__________________
How much do you spend on Electricity monthly	__________________
How much do you spend on Paraffin monthly	__________________
How much do you spend on Coal monthly	__________________
How much do you spend on Wood monthly	__________________
How much do you spend on Gas	__________________
How much do you spend on other type of fuel monthly	__________________
Specify other type of fuel	__________________
How much do you spend on Alcohol monthly	__________________
How much do you spend on Cigarettes monthly	__________________
How much do you spend on phone monthly	__________________
How much do you spend on medical expenses monthly	__________________
How much do you spend on Schooling or University monthly	__________________
How much do you spend on debt repayment monthly	__________________
How much do you spend on entertainment monthly (movies etc.)	__________________
Specify type of entertainment	__________________
Do you have medical aid?	○ Yes<br>○ No

Where do you get your food from? (choose all that apply)	☐ Grow your own food<br>☐ Buy food<br>☐ From charities<br>☐ From other waste pickers you work with<br>☐ From landfill site<br>☐ Other
If other, please explain	__________________________________

Food security (Community Child Hunger Identification Project Index - CCHIP)

Has your household ever run out of money to buy food in the past 30 days?	○ Yes<br>○ No
Has your household ever run out of money to buy food 5 or more days in the past 30 days?	○ Yes<br>○ No
Ever rely on limited number of foods to feed children because you are running out of money for food in the past 30 days?	○ Yes<br>○ No
Ever rely on limited foods because you ran out of money for food 5 or more days in past 30 days?	○ Yes<br>○ No
Ever cut meal size or skip meals because there is not enough money for food in past 30 days?	○ Yes<br>○ No
Ever cut meal size or skip meals because there is not enough money for food in 5 or more days in past 30 days?	○ Yes<br>○ No
Ever eat less because there is not enough food in the past 30 days?	○ Yes<br>○ No
Ever eat less because there is not enough food in 5 or more days in the past 30 days?	○ Yes<br>○ No
Do your children ever eat less because there is not enough money in the past 30 days?	○ Yes<br>○ No
Do your children ever eat less because there is not enough in 5 or more days in the past 30 days?	○ Yes<br>○ No
Do your children ever say they are hungry because there is not enough food in the house in the past 30 days ?	○ Yes<br>○ No
Do your children ever say they are hungry because not enough food in the house 5 or more days in the past 30 days ?	○ Yes<br>○ No
Ever cut meal size of children or skip meals because not enough money to buy food in past 30 days?	○ Yes<br>○ No

Ever cut meal size of children or skip meals because not enough money to buy food 5 or more days in past 30 days?	◯ Yes<br>◯ No
Do any of children ever go to bed hungry because not enough money for food in the past 30 days?	◯ Yes<br>◯ No
Do your children go to bed hungry because not enough money for food in 5 or more days in past 30 days?	◯ Yes<br>◯ No

Occupational history

Apart from collecting recyclable material and getting grants, do you get money from any other sources?	◯ Yes<br>◯ No
If yes, what are these other sources of income? Please list them	__________________________________
How much do you make in total from your other sources of income?	____________________________
Before working as a waste picker, what was your previous type of employment(s)	__________________________________
What was the reason for leaving your last job?	__________________________________
For how many years have you worked as a waste picker?	____________________________
How many days per week do you work on the landfill?	____________________________
How many hours per day are you on the landfill?	____________________________
On average, how many hours do you work per day?	____________________________

Waste Picking

Why did you choose waste picking?	__________________________________
What do you like about waste picking?	__________________________________
What don't you like about waste picking?	__________________________________

Do you collect any of the following waste? (choose all that apply)	☐ Paper or cardboard<br>☐ Plastic<br>☐ Metals or cans<br>☐ Electronic waste<br>☐ glass<br>☐ cloth<br>☐ other
If other, please specify...	__________________________________
If you collect more than one type of material, which material do you collect the most of?	◯ Paper or cardboard<br>◯ Plastic<br>◯ Metals or cans<br>◯ Electronic waste<br>◯ glass<br>◯ cloth<br>◯ other
If other, please specify...	__________________________________
Do you only collect one type of waste per day?	◯ Yes<br>◯ No
How much money do you earn from waste picking on a good day?	__________________________________
How much money do you earn from waste picking on a bad day?	__________________________________
What amount of waste do you remove on average per day, using a refuse black bag as reference?	__________________________________
How much does it weigh on average per day?	__________________________________
How do you transport your materials to the next location?	__________________________________
How often do you take the material to sell? (choose one)	◯ Daily<br>◯ Twice weekly<br>◯ Weekly<br>◯ 2 weeks<br>◯ Monthly<br>◯ Other
If other, explain...	__________________________________
Where do you take your waste to sell?	☐ Directly to the recycling centre<br>☐ To dealers who will sell to recycling centre<br>☐ Other
If other, elaborate	__________________________________

If you don't sell your waste everyday, where do you store the waste you have collected?	☐ At home<br>☐ On this landfill<br>☐ On another landfill<br>☐ Other
If other, please elaborate...	__________________________
Do you have access to toilet facilities at the landfill?	◯ Yes<br>◯ No
If no, what do you do?	__________________________
Do you think washing hands is important?	◯ Yes<br>◯ No
Do you have access to water on the landfill?	◯ Yes<br>◯ No
Do you eat on the landfill while working?	◯ Yes<br>◯ No
Do you wash your hands before eating while working on the landfill site?	◯ Yes<br>◯ No
Do you wash your hands at the end of the day, after work?	◯ Yes<br>◯ No
What do you use to wash your hands? (choose all that apply)	☐ Water<br>☐ Water and soap<br>☐ Other
If other, what?	__________________________
What do you use to protect yourself with while working?	☐ None<br>☐ Masks<br>☐ Gloves<br>☐ Boots or closed shoes<br>☐ Other
If other, please name	__________________________
If yes, where did you get it from?	__________________________
Do you wear them while working?	◯ Always<br>◯ Sometimes<br>◯ Never
If never, why not?	__________________________

Occupational Exposures

Do you lift heavy objects while working on the landfill?	◯ Yes<br>◯ No
Have you ever been cut by the materials you handle on the landfill site?	◯ Yes<br>◯ No
Have you ever handled needles on the landfill site?	◯ Yes<br>◯ No
Have you ever been injured by a needle on the landfill site?	◯ Yes<br>◯ No
Have you ever handled blood on the landfill site?	◯ Yes<br>◯ No
Are there dogs on the landfill site?	◯ Yes<br>◯ No
Have you ever been attacked by a dog on the landfill site?	◯ Yes<br>◯ No
Have you seen rats/mice on the landfill site?	◯ Yes<br>◯ No
Have you ever been bitten by rodents on the landfill site?	◯ Yes<br>◯ No
How problematic are mosquitoes on the landfill site?	◯ No problem<br>◯ Moderate problem<br>◯ Major problem
How problematic is airborne dust on the landfill site?	◯ No problem<br>◯ Moderate problem<br>◯ Major problem
How problematic are strong gas smells on the landfill site?	◯ No problem<br>◯ Moderate problem<br>◯ Major problem
Are there pools of dirty water on the surface of the landfill site?	◯ Yes<br>◯ No
Have you ever had waste fall on top of you?	◯ Yes<br>◯ No
Have you ever been hurt by waste falling on you?	◯ Yes<br>◯ No
Have you ever hurt yourself by falling on the landfill site?	◯ Yes<br>◯ No
have you ever seen a fire on the landfill site?	◯ Yes<br>◯ No

have you ever seen an explosion on the landfill site?	◯ Yes<br>◯ No
have you ever been injured by any type of vehicle on the landfill site?	◯ Yes<br>◯ No
Have you ever handled paint on the landfill site?	◯ Yes<br>◯ No
Have you ever handled chemicals, such as detergents or anything that smells very strong?	◯ Yes<br>◯ No
Have you ever been injured during violence involving another waste picker?	◯ Yes<br>◯ No
Have you ever been injured during violence involving security guards?	◯ Yes<br>◯ No
What 3 or more things that worry you the most about working on the landfill?	__________________________________
Do you think that the landfill site is a safe place to work?	◯ Yes<br>◯ No<br>◯ Not sure
Do you experience any problems accessing the landfill site?	◯ Yes<br>◯ No
If your answer is Yes, please explain...	__________________________________
How do security guards at the landfill treat you?	__________________________________
Do you belong to any waste picker forum?	◯ Yes<br>◯ No
If Yes, in what ways does belonging to the forum help you?	__________________________________

Health

Do you smoke currently?	◯ Yes<br>◯ No
If No, have you ever smoked?	◯ Yes<br>◯ No

If Yes, what do you smoke?	☐ Cigarettes<br>☐ Snuff<br>☐ Chewing tobacco<br>☐ Dagga/weed<br>☐ Hookah<br>☐ Other
If you smoke other, Please explain...	__________________________________
How much do you smoke/chew or snuff per day?	__________________________________
For how many years have you smoked?	__________________________________
How often do you have a drink containing alcohol?	◯ Never<br>◯ Monthly/less<br>◯ 2 to 4 times a month<br>◯ 2 to 3 times a week<br>◯ 4 or more times a week
How many drinks containing alcohol do you have on a typical day when you are drinking?	◯ 1 or 2<br>◯ 3 or 4<br>◯ 5 or 6<br>◯ 7 or 8 or 9<br>◯ 10 or more
How often do you have six or more drinks on one occasion?	◯ Never<br>◯ less than monthly<br>◯ Monthly<br>◯ Weekly<br>◯ Daily or almost daily
How often during the last year have you found that you were unable to stop drinking once started?	◯ Never<br>◯ less than monthly<br>◯ Monthly<br>◯ Weekly<br>◯ Daily or almost daily
How often during last year have you failed to do what was expected from you due to drinking?	◯ Never<br>◯ less than monthly<br>◯ Monthly<br>◯ Weekly<br>◯ Daily or almost daily
How often during the last year have you needed a first drink in morning after a heavy drinking session?	◯ Never<br>◯ less than monthly<br>◯ Monthly<br>◯ Weekly<br>◯ Daily or almost daily
How often during the last year have you had a feeling of guilt or remorse after drinking?	◯ Never<br>◯ less than monthly<br>◯ Monthly<br>◯ Weekly<br>◯ Daily or almost daily

How often during the last year have you been unable to remember night before due to drinking?	◯ Never<br>◯ less than monthly<br>◯ Monthly<br>◯ Weekly<br>◯ Daily or almost daily
have you or someone else been injured as a result of your drinking in the last year?	◯ No<br>◯ Yes<br>◯ True but not in the last year
Has a relative or a friend or a doctor or another health worker been concerned about your drinking?	◯ No<br>◯ Yes<br>◯ True but not in the last year
In general, would you say your health is...	◯ Excellent<br>◯ Very good<br>◯ Good<br>◯ Fair<br>◯ Poor
If fair or poor, please explain...	__________________________________
In the past 2 weeks, have you experienced Cough?	◯ Yes<br>◯ No
If you experienced cough, where did you seek treatment	◯ Clinic<br>◯ Doctor<br>◯ Traditional healer<br>◯ Self medicated<br>◯ Other<br>◯ No treatment sought
If treatment for cough was sought at other, please explain	____________________________
In the past 2 weeks, have you experienced Itchy rash?	◯ Yes<br>◯ No
If you experienced Itchy rash in the past 2 weeks, where did you seek treatment?	◯ Clinic<br>◯ Doctor<br>◯ Traditional healer<br>◯ Self-medicated<br>◯ Other<br>◯ No treatment sought
If treatment for itchy rash was sought at other, please name	____________________________
In the past 2 weeks, have you experienced runny/blocked nose?	◯ Yes<br>◯ No

If you experienced runny/blocked nose in the past 2 weeks, where did you seek treatment?	◯ Clinic<br>◯ Doctor<br>◯ Traditional healer<br>◯ Self medicated<br>◯ Other<br>◯ No treatment sought
If treatment for runny/blocked nose was sought at other, please name	__________________________________
In the past 2 weeks, have you experienced teary/watery eyes?	◯ Yes<br>◯ No
If you experienced teary/watery eyes in the past 2 weeks, where did you seek treatment?	◯ Clinic<br>◯ Doctor<br>◯ Traditional healer<br>◯ Self medicated<br>◯ Other<br>◯ No treatment sought
If treatment for teary/watery eyes was sought at other, please name	__________________________________
In the past 2 weeks, have you experienced sneezing?	◯ Yes<br>◯ No
If you experienced sneezing in the past 2 weeks, where did you seek treatment?	◯ Clinic<br>◯ Doctor<br>◯ Traditional healer<br>◯ Self-medicated<br>◯ Other<br>◯ No treatment sought
If treatment for sneezing was sought at other, please name	__________________________________
In the past 2 weeks, have you experienced breathlessness?	◯ Yes<br>◯ No
If you experienced breathlessness in the past 2 weeks, where did you seek treatment?	◯ Clinic<br>◯ Doctor<br>◯ Traditional healer<br>◯ Self-medicated<br>◯ Other<br>◯ No treatment sought
If treatment for breathlessness was sought at other, please name	__________________________________
In the past 2 weeks, have you experienced rapid breathing?	◯ Yes<br>◯ No
If you experienced rapid breathing in the past 2 weeks, where did you seek treatment?	◯ Clinic<br>◯ Doctor<br>◯ Traditional healer<br>◯ Self medicated<br>◯ Other<br>◯ No treatment sought

If treatment for rapid breathing was sought at other, please name	__________________________________
In the past 2 weeks, have you experienced rapid heart rate?	◯ Yes<br>◯ No
If you experienced rapid heart rate in the past 2 weeks, where did you seek treatment?	◯ Clinic<br>◯ Doctor<br>◯ Traditional healer<br>◯ Self medicated<br>◯ Other<br>◯ No treatment sought
If treatment for rapid heart rate was sought at other, please name	__________________________________
In the past 2 weeks, have you experienced nausea/vomiting?	◯ Yes<br>◯ No
If you experienced nausea/vomiting in the past 2 weeks, where did you seek treatment?	◯ Clinic<br>◯ Doctor<br>◯ Traditional healer<br>◯ Self medicated<br>◯ Other<br>◯ No treatment sought
If treatment for nausea/vomiting was sought at other, please name	__________________________________
In the past 2 weeks, have you experienced diarrhoea?	◯ Yes<br>◯ No
If you experienced diarrhoea in the past 2 weeks, where did you seek treatment?	◯ Clinic<br>◯ Doctor<br>◯ Traditional healer<br>◯ Self medicated<br>◯ Other<br>◯ No treatment sought
If treatment for diarrhoea was sought at other, please name	__________________________________
In the past 2 weeks, have you experienced headache?	◯ Yes<br>◯ No
If you experienced headache in the past 2 weeks, where did you seek treatment?	◯ Clinic<br>◯ Doctor<br>◯ Traditional healer<br>◯ Self-medicated<br>◯ Other<br>◯ No treatment sought
If treatment for headache was sought at other, please name	__________________________________
In the past 2 weeks, have you experienced loss of coordination?	◯ Yes<br>◯ No

If you experienced loss of coordination in the past 2 weeks, where did you seek treatment?	◯ Clinic<br>◯ Doctor<br>◯ Traditional healer<br>◯ Self medicated<br>◯ Other<br>◯ No treatment sought
If treatment for loss of coordination was sought at other, please name	__________________________
In the past 2 weeks, have you experienced fever?	◯ Yes<br>◯ No
If you experienced fever in the past 2 weeks, where did you seek treatment?	◯ Clinic<br>◯ Doctor<br>◯ Traditional healer<br>◯ Self medicated<br>◯ Other<br>◯ No treatment sought
If treatment for fever was sought at other, please name	__________________________
In the past 2 weeks, have you experienced muscle aches?	◯ Yes<br>◯ No
If you experienced muscle aches in the past 2 weeks, where did you seek treatment?	◯ Clinic<br>◯ Doctor<br>◯ Traditional healer<br>◯ Self medicated<br>◯ Other<br>◯ No treatment sought
If treatment for muscle aches was sought at other, please name	__________________________
In the past 2 weeks, have you experienced dizziness?	◯ Yes<br>◯ No
If you experienced dizziness in the past 2 weeks, where did you seek treatment?	◯ Clinic<br>◯ Doctor<br>◯ Traditional healer<br>◯ Self medicated<br>◯ Other<br>◯ No treatment sought
If treatment for dizziness was sought at other, please name	__________________________
In the past 2 weeks, have you experienced sunburn?	◯ Yes<br>◯ No
If you experienced sunburn in the past 2 weeks, where did you seek treatment?	◯ Clinic<br>◯ Doctor<br>◯ Traditional healer<br>◯ Self medicated<br>◯ Other<br>◯ No treatment sought

If treatment for sunburn was sought at other, please name	__________________

Chronic Illness:

Do you have now, or have you ever been diagnosed with Diabetes?	◯ Yes<br>◯ No
If you have diabetes, where did you seek treatment?	◯ Clinic<br>◯ Doctor<br>◯ Traditional healer<br>◯ Self-medicated<br>◯ Other<br>◯ No treatment sought
If treatment for diabetes was sought at other, please name	__________________
Do you have now, or have you ever been diagnosed with hypertension?	◯ Yes<br>◯ No
If you have Hypertension, where did you seek treatment?	◯ Clinic<br>◯ Doctor<br>◯ Traditional healer<br>◯ Self-medicated<br>◯ Other<br>◯ No treatment sought
If treatment for Hypertension was sought at other, please name	__________________
Do you have now, or have you ever been diagnosed with stroke?	◯ Yes<br>◯ No
If you have been diagnosed with stroke, where did you seek treatment?	◯ Clinic<br>◯ Doctor<br>◯ Traditional healer<br>◯ Self medicated<br>◯ Other<br>◯ No treatment sought
If treatment for stroke was sought at other, please name	__________________
Do you have now, or have you ever been diagnosed with asthma?	◯ Yes<br>◯ No
If you have been diagnosed with Asthma, where did you seek treatment?	◯ Clinic<br>◯ Doctor<br>◯ Traditional healer<br>◯ Self medicated<br>◯ Other<br>◯ No treatment sought
If treatment for Asthma was sought at other, please name	__________________

Do you have now, or have you ever been diagnosed with problems with vision?	◯ Yes<br>◯ No
If you have problems with Vision, where did you seek treatment?	◯ Clinic<br>◯ Doctor<br>◯ Traditional healer<br>◯ Self-medicated<br>◯ Other<br>◯ No treatment sought
If treatment for Vision was sought at other, please name	__________________________
Do you have now, or have you ever been diagnosed with problems with hearing?	◯ Yes<br>◯ No
If you have problems with Hearing, where did you seek treatment?	◯ Clinic<br>◯ Doctor<br>◯ Traditional healer<br>◯ Self medicated<br>◯ Other<br>◯ No treatment sought
If treatment for Hearing was sought at other, please name	__________________________
Do you have now, or have you ever been diagnosed with HIV?	◯ Yes<br>◯ No
If you have ever been diagnosed with HIV, where did you seek treatment?	◯ Clinic<br>◯ Doctor<br>◯ Traditional healer<br>◯ Self medicated<br>◯ Other<br>◯ No treatment sought
If treatment for HIV was sought at other, please name	__________________________
Do you have now, or have you ever been diagnosed with TB?	◯ Yes<br>◯ No
If you have ever been diagnosed with TB, where did you seek treatment?	◯ Clinic<br>◯ Doctor<br>◯ Traditional healer<br>◯ Self medicated<br>◯ Other<br>◯ No treatment sought
If treatment for TB was sought at other, please name	__________________________
Do you have now, or have you ever been diagnosed with mental illness?	◯ Yes<br>◯ No

If you have ever been diagnosed with mental illness, where did you seek treatment?	◯ Clinic<br>◯ Doctor<br>◯ Traditional healer<br>◯ Self medicated<br>◯ Other<br>◯ No treatment sought
If treatment for mental illness was sought at other, please name	__________________________
Do you have now, or have you ever been diagnosed with cancer?	◯ Yes<br>◯ No
If you have ever been diagnosed with cancer, where did you seek treatment?	◯ Clinic<br>◯ Doctor<br>◯ Traditional healer<br>◯ Self medicated<br>◯ Other<br>◯ No treatment sought
If treatment for cancer was sought at other, please name	__________________________
Do you have now, or have you ever been diagnosed with some type of disability?	◯ Yes<br>◯ No
If yes, where did you seek medical treatment?	☐ Clinic<br>☐ Doctor<br>☐ Traditional healer<br>☐ Self medicated<br>☐ Other
If treatment for disability was sought at other, please name	__________________________
In the past 12 months, have you had a persistent cough for more than three weeks?	◯ Yes<br>◯ No
If yes, how long did the coughing last?	__________________________
Have you ever had wheezing or whistling in the chest in the last 12 months?	◯ Yes<br>◯ No
If yes, how many attacks did you have in the last 12 months?	__________________________
Did you seek medical attention?	◯ Yes<br>◯ No
If yes, where did you go?	☐ Clinic<br>☐ Hospital<br>☐ Doctor<br>☐ Traditional healer<br>☐ Self-medicated<br>☐ Other

If other, please elaborate further...	__________________________________
What was the diagnosis?	____
Are there times when something at the landfill in the environment affects your chest?	◯ Yes<br>◯ No
If yes, how often have you experienced this?	◯ Everyday<br>◯ Several times a week<br>◯ About once a week<br>◯ Once or twice a month<br>◯ A few times in the past 6 months
If you know what it is please name it	____
Have you had an itchy rash that was coming and going for at least 6 months?	◯ Yes<br>◯ No
Have you had this rash any time in the last 6 months?	◯ Yes<br>◯ No
Has this rash affected folds of elbow?	◯ Yes<br>◯ No
Has this rash affected behind knees?	◯ Yes<br>◯ No
Has this rash affected in front of ankles?	◯ Yes<br>◯ No
Has this rash affected under buttocks?	◯ Yes<br>◯ No
Has this rash affected around neck, ears or eyes?	◯ Yes<br>◯ No
Did you seek medical treatment for your skin rash?	◯ Yes<br>◯ No
Where did you go?	☐ Clinic<br>☐ Hospital<br>☐ Doctor<br>☐ Traditional healer<br>☐ Self-medicated<br>☐ Other
Explain other	____

Please state the diagnosis?	__________________________
Have you ever been diagnosed with eczema by a doctor or nurse?	◯ Yes<br>◯ No
Have you experienced diarrhoea, nausea or vomiting?	◯ Yes<br>◯ No
If yes, how often in the past 6 months have you experienced this?	◯ Everyday<br>◯ Several times a week<br>◯ About once a week<br>◯ Once or twice a month<br>◯ A few times in the past 6 months
Did you seek medical treatment?	◯ Yes<br>◯ No
Who provided the medical treatment?	◯ Clinic<br>◯ Doctor<br>◯ Traditional healer<br>◯ Self medicated<br>◯ Other
Have you ever been injured at work in the last 6 months(e.g. cuts, fracture, sprains and/or burns)?	◯ Yes<br>◯ No
Fractures?	◯ Yes<br>◯ No
If yes, how often?	◯ Everyday<br>◯ Several times a week<br>◯ About once a week<br>◯ Once or twice a month<br>◯ A few times in the past 6 months
Who provided treatment?	◯ Pikitup staff<br>◯ Clinic<br>◯ Doctor<br>◯ Traditional healer<br>◯ self-medicated<br>◯ No treatment<br>◯ Other
Sprains and muscle strains?	◯ Yes<br>◯ No
How often?	◯ Everyday<br>◯ Several times a week<br>◯ About once a week<br>◯ Once or twice a month<br>◯ A few times in the past 6 months

Who provided the treatment?	◯ Pikitup staff<br>◯ Clinic<br>◯ Doctor<br>◯ Traditional healer<br>◯ self-medicated<br>◯ No treatment<br>◯ Other
Injured by landfill truck?	◯ Yes<br>◯ No
If injured by landfill truck, how often?	◯ Everyday<br>◯ Several times a week<br>◯ About once a week<br>◯ Once or twice a month<br>◯ A few times in the past 6 months
And who provided treatment?	◯ Pikitup staff<br>◯ Clinic<br>◯ Doctor<br>◯ Traditional healer<br>◯ self-medicated<br>◯ No treatment<br>◯ Other
Burns?	◯ Yes<br>◯ No
how frequent?	◯ Everyday<br>◯ Several times a week<br>◯ About once a week<br>◯ Once or twice a month<br>◯ A few times in the past 6 months
Who administered treatment	◯ Pikitup staff<br>◯ Clinic<br>◯ Doctor<br>◯ Traditional healer<br>◯ self-medicated<br>◯ No treatment<br>◯ Other
Hit by falling objects	◯ Yes<br>◯ No
How often are you hit by falling objects?	◯ Everyday<br>◯ Several times a week<br>◯ About once a week<br>◯ Once or twice a month<br>◯ A few times in the past 6 months
and who administered treatment?	◯ Pikitup staff<br>◯ Clinic<br>◯ Doctor<br>◯ Traditional healer<br>◯ self-medicated<br>◯ No treatment<br>◯ Other

Slips, trips and falls?	◯ Yes<br>◯ No
how often do you experience slips, trips and falls?	◯ Everyday<br>◯ Several times a week<br>◯ About once a week<br>◯ Once or twice a month<br>◯ A few times in the past 6 months
Who gave treatment?	◯ Pikitup staff<br>◯ Clinic<br>◯ Doctor<br>◯ Traditional healer<br>◯ self-medicated<br>◯ No treatment<br>◯ Other
Cuts and lacerations?	◯ Yes<br>◯ No
How often do you experience cuts and lacerations?	◯ Everyday<br>◯ Several times a week<br>◯ About once a week<br>◯ Once or twice a month<br>◯ A few times in the past 6 months
And who gave treatment?	◯ Pikitup staff<br>◯ Clinic<br>◯ Doctor<br>◯ Traditional healer<br>◯ self-medicated<br>◯ No treatment<br>◯ Other
Inhaling toxic fumes?	◯ Yes<br>◯ No
how often do you inhale these fumes?	◯ Everyday<br>◯ Several times a week<br>◯ About once a week<br>◯ Once or twice a month<br>◯ A few times in the past 6 months
Who provided treatment when you needed one?	◯ Pikitup staff<br>◯ Clinic<br>◯ Doctor<br>◯ Traditional healer<br>◯ self-medicated<br>◯ No treatment<br>◯ Other
exposure to sudden loud noise?	◯ Yes<br>◯ No
How often are you exposed to sudden loud noise?	◯ Everyday<br>◯ Several times a week<br>◯ About once a week<br>◯ Once or twice a month<br>◯ A few times in the past 6 months

Who provides treatment?	◯ Pikitup staff<br>◯ Clinic<br>◯ Doctor<br>◯ Traditional healer<br>◯ self-medicated<br>◯ No treatment<br>◯ Other
Other types of injury?	◯ Yes<br>◯ No
Please name other types	__________________________________
How often does this happen?	◯ Everyday<br>◯ Several times a week<br>◯ About once a week<br>◯ Once or twice a month<br>◯ A few times in the past 6 months
Who provided treatment when this happened?	◯ Pikitup staff<br>◯ Clinic<br>◯ Doctor<br>◯ Traditional healer<br>◯ self-medicated<br>◯ No treatment<br>◯ Other
If yes, please specify the type of injury...	◯ Fractures<br>◯ Sprains and muscle strains<br>◯ Injured by landfill truck<br>◯ Burns<br>◯ Being hit by falling objects<br>◯ trips and falls<br>◯ Cuts and lacerations<br>◯ Inhaling toxic fumes<br>◯ Exposure to sudden loud noise<br>◯ Other
If yes to 91.1, 91.3 or 91.4, please describe how you sustained your injury or injuries?	__________________________________
In the past 12 months, have you suffered from any body pains?	◯ Yes<br>◯ No
If yes, which of the following body pain?	☐ Back pain<br>☐ Joint pain<br>☐ Stiffness<br>☐ Other
If other body pains please specify:	____________________________
Did you seek medical treatment for your body pain?	◯ Yes<br>◯ No

Who offered the medical treatment?	◯ Clinic<br>◯ Doctor<br>◯ Traditional healer<br>◯ Self medicated<br>◯ Other
Do you have difficulty hearing or understanding people around you?	◯ Yes<br>◯ No
If yes, did you have a hearing problem before working on the landfill?	◯ Yes<br>◯ No
Is there a history of hearing problems or deafness in your family?	◯ Yes<br>◯ No
Have you ever been diagnosed with any of the following?	☐ No<br>☐ Ear infection<br>☐ Meningitis<br>☐ TB<br>☐ Mumps/measles
In the past 12 months, have you missed work due to any illness or injury?	◯ Yes<br>◯ No
If yes, please specify the illness or injury	______________________________
Do you think your illness or injury was related to your work?	◯ Yes<br>◯ No
If yes, please give details	______________________________
how many times have you been pregnant?	◯ 0<br>◯ 1<br>◯ 2<br>◯ 3<br>◯ 4<br>◯ 5<br>◯ 6<br>◯ 7<br>◯ 8<br>◯ 9<br>◯ 10<br>◯ 11<br>◯ 12<br>◯ 13<br>◯ 14<br>◯ 15

How many children do you have?	◯ 0<br>◯ 1<br>◯ 2<br>◯ 3<br>◯ 4<br>◯ 5<br>◯ 6<br>◯ 7<br>◯ 8<br>◯ 9<br>◯ 10<br>◯ 11<br>◯ 12<br>◯ 13<br>◯ 14<br>◯ 15
Have you ever had a miscarriage?	◯ Yes<br>◯ No
If yes, when did it happen?	◯ Before working as a waste picker<br>◯ While working as a waste picker
If yes, how many miscarriages?	______________________________
Have you ever had a still birth?	◯ Yes<br>◯ No
If yes, when did this happen?	◯ Before working as a waste picker<br>◯ While working as a waste picker
If yes, how many still births?	______________________________
Was your last/ most recent pregnancy expected or not a surprise?	◯ Yes<br>◯ No
How long did it take you to fall pregnant?	◯ Years<br>◯ Months<br>◯ Don't know
Were you using contraceptives before you fell pregnant?	◯ Yes<br>◯ No
If yes, when did you stop your contraceptives?	◯ Years<br>◯ Months<br>◯ Don't know
Why did you stop your contraceptives?	◯ Fell pregnant<br>◯ Side effects<br>◯ Cost<br>◯ Religion<br>◯ N/A<br>◯ Other

What was the outcome of this pregnancy?	◯ Miscarriage<br>◯ Still birth<br>◯ Live birth
How many weeks were you when you delivered your last baby?	__________________________
What was the birth weight of you most recent child?	__________________________

Mental Health Screening

Do you often have headaches?	◯ Yes<br>◯ No
Is your appetite poor?	◯ Yes<br>◯ No
Do you sleep badly?	◯ Yes<br>◯ No
Are you easily frightened?	◯ Yes<br>◯ No
Do your hands shake?	◯ Yes<br>◯ No
Do you feel nervous, tense or worried?	◯ Yes<br>◯ No
Is your digestion poor?	◯ Yes<br>◯ No
Do you have trouble thinking clearly?	◯ Yes<br>◯ No
Do you feel unhappy?	◯ Yes<br>◯ No
Do you cry more than usual?	◯ Yes<br>◯ No
Do you find it difficult to enjoy your daily activities?	◯ Yes<br>◯ No
Do you find it difficult to make decisions?	◯ Yes<br>◯ No
Is your daily work suffering?	◯ Yes<br>◯ No
Are you unable to play a useful part in life?	◯ Yes<br>◯ No

Have you lost interest in things?	◯ Yes<br>◯ No
Do you feel that you are a worthless person?	◯ Yes<br>◯ No
Has the thought of ending your life been on your mind?	◯ Yes<br>◯ No
Do you feel tired all the time?	◯ Yes<br>◯ No
Do you have uncomfortable feelings in your stomach?	◯ Yes<br>◯ No
Are you tired easily?	◯ Yes<br>◯ No
In the last 12 months, did you go to a clinic or hospital to see a doctor or nurse?	◯ Yes<br>◯ No
Do you attend the local clinic?	◯ Where you live<br>◯ Where you work<br>◯ Any
Do you always go to the same clinic?	◯ Yes<br>◯ No
If no, please explain	__________________________________
What obstacles or barriers do you face when needing to access health care services?	☐ Transport problems<br>☐ Unable to pay for services<br>☐ Unable to take time off work during the day<br>☐ No services available in the community where I live<br>☐ No services available close to where I work<br>☐ Have problems getting child care<br>☐ Language problems<br>☐ Went to the clinic, but was turned away<br>☐ Poor quality of services or care<br>☐ Long waiting list to see the doctor or nurse<br>☐ Other
If other obstacles, please name them.	__________________________________
What would you consider as the major obstacle you faced when accessing health care services?	__________________________________
Do you feel that you are treated well at the clinic or hospital?	◯ Yes<br>◯ No
Do you feel that you are treated differently or discriminated against at the clinic or hospital?	◯ Yes<br>◯ No

Please explain your answer	__________________________________
have you ever been admitted to a hospital?	◯ Yes<br>◯ No
If yes, when were you admitted and what was the diagnosis?	__________________________________
How would you rate your quality of life?	◯ Very poor<br>◯ Poor<br>◯ Neither poor nor good<br>◯ Good<br>◯ Very good
How satisfied are you with your health?	◯ Very dissatisfied<br>◯ Dissatisfied<br>◯ Neither satisfied nor dissatisfied<br>◯ Satisfied<br>◯ Very satisfied
How satisfied are you with your access to health care services?	◯ Very dissatisfied<br>◯ Dissatisfied<br>◯ Neither satisfied nor dissatisfied<br>◯ Satisfied<br>◯ Very satisfied
How satisfied are you with your capacity to work?	◯ Very dissatisfied<br>◯ Dissatisfied<br>◯ Neither satisfied nor dissatisfied<br>◯ Satisfied<br>◯ Very satisfied
To what extent do you feel that physical pain prevents you from doing what you need to do?	◯ Not at all<br>◯ A little<br>◯ A moderate amount<br>◯ Very much<br>◯ An extreme amount
How much do you enjoy life?	◯ Not at all<br>◯ A little<br>◯ A moderate amount<br>◯ Very much<br>◯ An extreme amount
To what extent do you feel your life to be meaningful?	◯ Not at all<br>◯ A little<br>◯ A moderate amount<br>◯ Very much<br>◯ An extreme amount
How much do you fear the future?	◯ Not at all<br>◯ A little<br>◯ A moderate amount<br>◯ Very much<br>◯ An extreme amount

How much do you worry about your health?	◯ Not at all<br>◯ A little<br>◯ A moderate amount<br>◯ Very much<br>◯ An extreme amount
How much are you able to concentrate?	◯ Not at all<br>◯ A little<br>◯ A moderate amount<br>◯ Very much<br>◯ An extreme amount
How safe do you feel in your daily life?	◯ Not at all<br>◯ A little<br>◯ A moderate amount<br>◯ Very much<br>◯ An extreme amount
How healthy is your physical environment?	◯ Not at all<br>◯ A little<br>◯ A moderate amount<br>◯ Very much<br>◯ An extreme amount

Biochemistry test results

Systolic blood pressure	__________________________
Diastolic blood pressure	__________________________
Fasting blood glucose	__________________________
Haemoglobin	__________________________
Cholesterol	__________________________
